# Future Self-Continuity and Psychological Well-Being in Chinese College Students: The Mediating Role of Meaning in Life and the Moderating Role of Moral Identity

**DOI:** 10.3390/bs16050647

**Published:** 2026-04-26

**Authors:** Xiaowen Zhu, Guoliang Qu, Guanrui Wang, Quan Wei, Yangmei Luo

**Affiliations:** 1 School of Teacher Development, Shaanxi Normal University, Xi’an 710062, China; xiaowenzhu@snnu.edu.cn; 2 School of Tea and Food Science, Xinyang Normal University, Xinyang 464000, China; 3 School of Psychology, Shaanxi Normal University, Xi’an 710062, China; guoliangqu@snnu.edu.cn (G.Q.); wang_gr@snnu.edu.cn (G.W.); psychowei@snnu.edu.cn (Q.W.)

**Keywords:** future self-continuity, psychological well-being, meaning in life, moral identity, Chinese college students

## Abstract

Future self-continuity is crucial for mental health and social adaptation. Previous research has mainly focused on its direct influence on cognitive decision-making and goal pursuit, with little emphasis on its effect on psychological well-being. This study addressed the research gap by employing a questionnaire to examine the link between future self-continuity and psychological well-being in 890 Chinese college students, emphasizing the mediating effect of meaning in life and the moderating effect of moral identity. The findings indicated that future self-continuity directly predicts psychological well-being and indirectly contributes by enhancing meaning in life. Moral identity negatively moderated both pathways, making the positive impacts of future self-continuity on psychological well-being and meaning in life more pronounced in individuals with low moral identity and diminished in those with high moral identity. These findings highlight future self-continuity as a cognitive resource that fosters well-being by cultivating meaning in life, while also identifying moral identity as a boundary condition. This research reveals the mechanisms and boundary conditions linking future self-continuity to psychological well-being, offering actionable insights to enhance mental health in college students.

## 1. Introduction

The pursuit of happiness represents a lasting concern within human society. In the field of psychology, well-being refers to how favorably individuals judge their lives against their own standards (Diener, 2012). It serves not only to reflect subjective life satisfaction but also to capture individuals’ positive adaptive states across emotional, cognitive, and functional domains (Burns & Crisp, 2022). Individuals pursue happiness through two philosophical orientations: hedonism, focused on pleasure and comfort, and eudaimonism, centered on meaning and self-actualization (Luo et al., 2019). Numerous studies have confirmed that elevated well-being is closely related to positive mental health, harmonious social interactions, exceptional academic achievements, and enhanced psychological resilience (Bücker et al., 2018; Mauss et al., 2012; Diener et al., 2018; Yıldırım et al., 2025). The absence of well-being often results in psychological issues like anxiety and depression (Crego et al., 2021) and can increase the risk of physical illnesses (Steptoe, 2019).

Future self-continuity, as a key cognitive asset connecting the “present self” with the “future self” (Rutt & Löckenhoff, 2016), offers individuals a transcendent temporal perspective beyond the present. It enables individuals to integrate their daily experiences into a coherent life narrative (McAdams, 2013), thereby enhancing their perception of life goals and directions. According to “Identity-Based Motivation Theory” (IBM; Oyserman & Destin, 2010), current behavior is guided by the self-identity activated in a given situation. When the “future self” identity is activated, individuals are more motivated to act in ways consistent with that identity, which in turn promotes meaning-seeking and psychological well-being by fulfilling basic needs for coherence and purpose (Oyserman & Dawson, 2019). Moreover, Oyserman and Dawson (2019) note that individuals hold multiple self-identities simultaneously. When the moral identity is highly significant, the “moral self” may compete with the “future self”, thereby moderating the influence of future self-continuity on meaning in life and psychological well-being.

College students are at a critical stage of identity formation, where the core task is to integrate past, present, and future selves into a stable self-identity (Erikson, 1968). They tend to exhibit the developmental characteristics of seeking meaning in life and pursuing life goals (Lui et al., 2022), and their moral identity remains uncrystallized and considerable plasticity (Lefebvre et al., 2024). Research indicates that individuals around the age of 20 show similar and moderate levels of both the presence of meaning and search for meaning (Aftab et al., 2019). Given the Chinese cultural context of collectivism and long-term orientation (Hofstede, 2001), in which future self-continuity is particularly salient (Ji et al., 2019), psychological well-being has received comparatively limited attention in positive psychology compared with the relatively mature research on subjective well-being (Allen et al., 2021; Kaufman, 2023). Therefore, the present study focuses on Chinese college students to investigate the mechanism through which future self-continuity influences psychological well-being, with a particular emphasis on the mediating role of meaning in life and the moderating role of moral identity. This will not only provide a new theoretical perspective for understanding the relationship between future self-continuity and psychological well-being but also offer empirical evidence to inform future educational interventions.

### 1.1. Future Self-Continuity and Psychological Well-Being

Future self-continuity reflects the perceived psychological link between an individual’s current and anticipated selves (Rutt & Löckenhoff, 2016). It is a key cognitive variable that drives individuals’ long-term behavior. Individuals with greater future self-continuity are more likely to make present decisions that favor their future. This construct has been demonstrated to serve important functions in multiple domains, including long-term adaptive health behaviors (Fry & Debats, 2011), intertemporal choice (Hershfield, 2011), temporal discounting (Bartels & Urminsky, 2011), academic achievement (Adelman et al., 2017), and self-evaluation (Chu & Lowery, 2024).

Psychological well-being is an important indicator of an individual’s state of psychological functioning in the process of fulfilling their potential and pursuing meaning in life. As conceptualized in Ryff’s (1989) model of psychological well-being, this construct is multidimensional, with personal growth and purpose in life being two core components. Unlike subjective well-being, psychological well-being centers on individuals’ long-term development and self-actualization. Individuals with elevated psychological well-being tend to frequently self-reflect, actively pursue and realize their true selves, and often exhibit a heightened sense of self-connectedness (Huta, 2012). Recognized as a fundamental marker of positive psychological functioning, psychological well-being serves not only to reflect individuals’ level of psychological adaptation but also to forecast their social adaptability and long-term developmental potential. It constitutes an important foundation for individuals to achieve healthy growth and comprehensive development.

A strong theoretical and empirical link has been established between future self-continuity and psychological well-being. Building upon future orientation theory, a clear, positive, and presently connected representation of the future self offers individuals a coherent motivational and meaning framework (Seginer, 2009). Individuals with greater future self-continuity can clearly envision their future selves, enhancing their certainty about life goals and perceived control over personal growth. This cognitive foundation enhances psychological well-being. Empirical evidence supports this proposition. Dai et al. (2023) conducted a cross-sectional study and observed a significant positive correlation between psychological well-being and global self-continuity, which refers to the perceived continuity linking past, present, and future selves. In a ten-year longitudinal investigation, Reiff et al. (2020) observed that individuals’ perceived similarity to their future selves significantly predicted life satisfaction a decade later. Sokol and Serper (2019) demonstrated through experimental research that participants exposed to a manipulation aimed at increasing future self-continuity experienced higher levels of happiness and positive affect than those in the control group. These findings indicate that future self-continuity could be a strong predictor of psychological well-being.

### 1.2. The Mediating Role of Meaning in Life

Meaning in life is suggested to be a vital connection between future self-continuity and psychological well-being. Defined as the perceived coherence, significance, and purposefulness of one’s existence (Costin & Vignoles, 2019), it comprises two main dimensions: the presence of meaning and the search for meaning (Steger et al., 2008b). Meaningfulness serves as a key marker of positive psychological functioning, buffering against stress (Arslan & Yıldırım, 2021) while also constituting a fundamental element of the psychological well-being framework (Ryff, 1989). Baumeister’s large-scale survey revealed that while happiness and meaning in life are closely related, they involve distinct psychological processes. Specifically, happiness is strongly tied to “getting what one wants and needs,” “immediate pleasure,” and “need satisfaction,” whereas meaning in life is strongly connected with “integrating past, present, and future,” “giving to others,” and “expressing one’s authentic self” (Baumeister et al., 2013). This ability to “integrate time” implies that future self-continuity may not directly provide immediate happiness, but rather aids individuals in constructing a coherent life narrative, thereby elevating the sense of meaning in life.

Mental simulation theory posits that individuals imbue their present actions with meaning and guide their decision-making by constructing mental representations of future scenarios (Gordon, 1986). Individuals with higher future self-continuity excel in simulations that actively facilitate meaning-making, enabling them to perceive the future value and purpose of their current actions (Waytz et al., 2015). A growing body of evidence indicates that future self-continuity fosters meaning in life through the pathway of authentic self-perception (Chu & Lowery, 2024; Hong et al., 2024).

The broaden-and-build theory of positive emotions (Fredrickson, 2001) suggests that positive emotions derived from a sense of meaning, such as contentment, serenity, gratitude, and hope, temporarily expand individuals’ cognitive-behavioral repertoires (e.g., cognitive openness, exploratory behavior, and social connectedness) and contribute to the long-term development of lasting personal resources like psychological resilience, knowledge and skills, and social support. Arslan and Yıldırım (2021) presented longitudinal evidence indicating that meaning in life significantly predicted individuals’ psychological well-being, even amid the substantial stress of the COVID-19 pandemic. Individuals with higher pre-pandemic meaning in life exhibited improved psychological well-being at follow-up. We propose that future self-continuity could enhance meaning in life by triggering “temporal integration” and “meaning-making” processes, thus fostering psychological well-being. Meaning in life mediates the relationship between future self-continuity and psychological well-being.

### 1.3. The Moderating Role of Moral Identity

While future self-continuity can improve individuals’ sense of meaning in life and psychological well-being, this beneficial effect may depend on the strength of their moral identity. Moral identity concerns the centrality of moral traits to self-concept (Aquino & Reed, 2002) and is bifurcated into internalization (their integration into the self) and symbolization (their public expression) (Aquino & Kay, 2018). Moral identity, viewed as a stable self-schema, can be understood through self-discrepancy theory regarding its moderating role. Self-discrepancy theory (Higgins, 1987) posits that the self-system comprises various representations. The “ideal self” encompasses desired attributes that inspire aspirations and positive endeavors, while the “ought self” involves attributes perceived as necessary, influenced by duties and moral standards. Future self-continuity primarily activates the “ideal self,” manifesting as individuals’ mental representations and positive evaluations of “who they desire to become” in the future. Moral identity aligns with the “ought self,” reflecting how individuals internalize moral traits as integral to their self-concept, guiding their adherence to moral standards. Individuals with strong moral identity align their aspirations and obligations with moral standards, enhancing the positive impact of future self-continuity.

A recent meta-analysis indicates that moral identity is positively linked to emotional well-being, with its connections to meaning in life and self-esteem characterized by moderate effect sizes (Goering et al., 2024). Individuals with a strong moral identity often demonstrate a higher alignment between their values and actions (Van Gils & Horton, 2019). Participating in moral actions not only yields positive emotional outcomes (Miles & Upenieks, 2022) but also enhances self-perception (McKinnon, 2005). Moral identity enriches individuals’ sense of meaning in life and well-being through three interrelated mechanisms: fostering increased moral engagement, sustaining a cohesive self-concept, and generating frequent positive emotions. Therefore, we propose that moral identity positively moderates the effect of future self-continuity on both meaning in life and psychological well-being.

### 1.4. The Present Study

**Figure 1:**
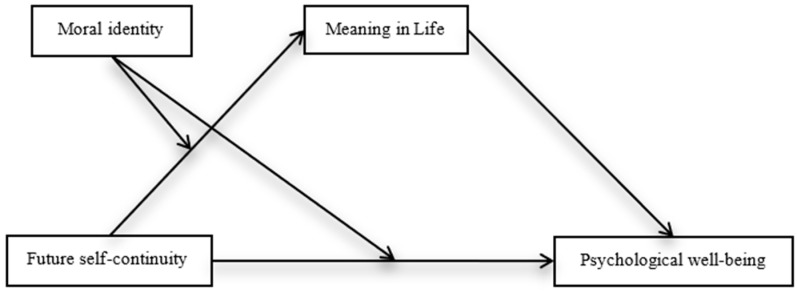
Proposed moderated mediation model.

The present study proposed the following hypotheses for Chinese college students based on the theoretical framework outlined above. Hypothesis 1: Future self-continuity positively influences psychological well-being. Hypothesis 2: Meaning in life mediates the relationship between future self-continuity and psychological well-being. Hypothesis 3: Moral identity positively moderates the relationship between future self-continuity and meaning in life. Hypothesis 4: Moral identity positively moderates the direct relationship between future self-continuity and psychological well-being. These predictions were tested via an integrated moderated mediation model (see Figure 1). The study seeks to advance understanding of the mechanisms and boundary conditions linking future self-continuity to psychological well-being, providing implications for mental health interventions targeting college populations.

## 2. Materials and Methods

### 2.1. Participants

Using a convenience sampling approach, college students were recruited from five provinces in China (Liaoning, Shaanxi, Anhui, Henan, and Shandong) via the online platform Wenjuanxing (Changsha Ranxing Information Technology Co., Ltd., Changsha, China). Prior to participation, all respondents were provided with information about the research purpose, data confidentiality protocols, and their right to voluntary participation. Each participant signed electronic informed consent before questionnaire administration. We distributed 1006 questionnaires. After excluding invalid responses, 890 valid questionnaires were retained (effective response rate = 88.47%). Responses were excluded based on the following criteria: (a) failure to pass two attention-check items; (b) completion time either too short or too long; and (c) patterned responding. The final sample comprised 320 male (35.96%) and 570 female (64.04%) participants, with a mean age of 19.04 years (*SD* = 1.12). Grade distribution was as follows: 463 freshmen (52.02%), 323 sophomores (36.29%), and 104 juniors (11.69%). In terms of academic discipline, 238 majored in humanities (26.74%), 424 in sciences (47.64%), and 228 in engineering (25.62%).

### 2.2. Measures

#### 2.2.1. Future Self-Continuity

Future self-continuity was evaluated using the Chinese version of the Future Self-Continuity Questionnaire, developed by Sokol and Serper (2019) and adapted by Shen et al. (2022). The instrument includes 10 items across three dimensions, with four items assessing similarity, such as “How similar are you now to the person you will be 10 years from now?” Vividness is assessed using three items, such as “How clearly can you envision yourself 10 years into the future?”, and positivity (3 items; “How much do you like the person you will be 10 years from now?”). Items are evaluated using a 7-point Likert scale, ranging from 1 (not at all similar/vivid/positive) to 7 (completely similar/vivid/positive). Item-level scores were averaged to create a composite score, with higher values denoting stronger future self-continuity. The scale’s Cronbach’s α coefficient in this study was 0.901.

#### 2.2.2. Meaning in Life

Meaning in life was evaluated using the Chinese version of the Meaning in Life Scale, developed by Steger et al. (2008b) and adapted by Liu and Gan (2010). This 9-item instrument comprises two subscales: The Presence of Meaning (five items; “I understand my life’s meaning”) and Search for Meaning (four items; “I am always looking to find my life’s purpose”). Items are evaluated on a 7-point Likert scale, where 1 indicates “strongly disagree” and 7 signifies “strongly agree”. Item scores were averaged, with higher values indicating a stronger sense of meaning in life. The scale’s Cronbach’s α coefficient in this study was 0.836.

#### 2.2.3. Moral Identity

Moral identity was evaluated using the Moral Identity Scale developed by Aquino and Reed (2002). The instrument comprises 10 items grouped into two dimensions: internalization (5 items; “Having these characteristics makes me feel good”) and symbolization (5 items; “I engage in activities that demonstrate these characteristics to others”). Responses are evaluated using a 5-point Likert scale, ranging from 1 (strongly disagree) to 5 (strongly agree). Item scores were averaged, with higher scores indicating a higher level of moral identity. The scale’s Cronbach’s α coefficient in this study was 0.768.

#### 2.2.4. Psychological Well-Being

To minimize participant fatigue, psychological well-being was gauged using the 18-item short form of Ryff’s Psychological Well-Being Scale (Ryff & Keyes, 1995). This instrument evaluates six dimensions: autonomy, environmental mastery, personal growth, positive relations with others, purpose in life, and self-acceptance, each measured by three items. Sample items include “I am easily influenced by people with strong opinions”, “In general, I feel I am in charge of my life”, and “I like most aspects of my personality”. Items are evaluated using a 6-point Likert scale anchored at 1 (strongly disagree) and 6 (strongly agree). Item scores were averaged to form a composite well-being score, with higher values reflecting greater psychological well-being. The scale’s Cronbach’s α coefficient in this study was 0.885.

#### 2.2.5. Procedure and Data Processing

The survey platform Wenjuanxing was set to require responses to all items before submission; consequently, no missing data occurred in this study. Descriptive statistics and correlation analyses were performed with SPSS 25.0 (IBM Corp., Armonk, NY, USA). Harman’s single-factor test (Podsakoff & Organ, 1986) was applied to evaluate common method bias. In addition, a confirmatory factor analysis (CFA) was conducted using Mplus 8.3 (Muthén & Muthén, Los Angeles, CA, USA) to provide a more rigorous test of common method bias (Podsakoff et al., 2003). The PROCESS macro for SPSS (version 4.0, Hayes, 2018) was utilized to perform moderated mediation analyses, with gender and age included as control variables. The significance of mediation and moderation effects was assessed using a bias-corrected percentile bootstrap method with 5000 resamples.

Because our research question focuses on overall pathways, future self-continuity and psychological well-being were retained as total scores, consistent with prior literature (Sokol & Serper, 2019; Xue et al., 2024). However, meaning in life and moral identity are theoretically multidimensional; therefore, we conducted separate moderated mediation analyses for their sub-dimensions.

## 3. Results

### 3.1. Common-Method Bias Test

The exclusive use of self-report measures in this study introduces a potential risk of common method bias. Harman’s single-factor test was conducted to investigate this issue. The analysis identified nine unrotated factors with eigenvalues exceeding one, explaining 67.93% of the total variance. The first factor explained 30.26% of the variance, which is below the recommended 40% threshold. To provide a more rigorous test, we conducted a confirmatory factor analysis (CFA) specifying a single factor loading all items (Podsakoff et al., 2003). This single-factor model showed very poor fit to the data: *χ*^2^ (1034) = 16,063.03, *p* < 0.001, CFI = 0.457, TLI = 0.432, RMSEA = 0.128 (90% CI = [0.126, 0.130]), SRMR = 0.127. The poor fit indicates that a single common factor cannot account for the majority of the variance, which argues against the presence of serious common method bias (Podsakoff et al., 2003). These results imply that common method bias does not pose a serious problem in the present study.

### 3.2. Preliminary Assessment

The means, standard deviations, and bivariate correlations among the study variables are displayed in Table 1. Correlation analysis revealed significant positive relationships among future self-continuity, meaning in life, moral identity, and psychological well-being, with correlation coefficients ranging from 0.38 to 0.64 (*rs* = 0.38~0.64, all *ps* < 0.001).

### 3.3. Analysis for the Proposed Model

All regression coefficients reported in Table 2 and Table 3 are unstandardized coefficients (denoted as *β* in the tables), as generated by the PROCESS macro (Hayes, 2018). First, with gender and age controlled, PROCESS Model 4 was used to examine the mediating role of meaning in life. Table 2 demonstrates that future self-continuity significantly predicts both meaning in life (*β* = 0.61, *p* < 0.001, 95% CI = [0.56, 0.66]) and psychological well-being (*β* = 0.13, *p* < 0.001, 95% CI = [0.07, 0.19]). Additionally, meaning in life significantly predicts psychological well-being (*β* = 0.28, *p* < 0.001, 95% CI = [0.22, 0.35]). The bias-corrected percentile bootstrap method revealed that meaning in life partially mediated the link between future self-continuity and psychological well-being. The study identified a direct effect of future self-continuity on psychological well-being of 0.13 (SE = 0.03, 95% CI = [0.07, 0.19]), an indirect effect of 0.17 (Boot SE = 0.03, 95% CI = [0.12, 0.22]), and a total effect of 0.30 (SE = 0.03, 95% CI = [0.25, 0.35]). The mediating effect represented 56.67% of the total effect. Thus, Hypotheses 1 and 2 received support.

Second, we conducted a moderated mediation analysis using PROCESS Model 8 (Hayes, 2018) to assess the moderating role of moral identity, controlling for gender and age. The findings (Table 3) indicated that future self-continuity significantly and positively predicted both meaning in life (*β* = 0.75, *p* < 0.001, 95% CI = [0.48, 1.03]) and psychological well-being (*β* = 0.48, *p* < 0.01, 95% CI = [0.20, 0.77]). The interaction between future self-continuity and moral identity significantly predicted meaning in life (*β* = −0.08, *p* < 0.05, 95% CI = [−0.16, −0.01]) and psychological well-being (*β* = −0.11, *p* < 0.01, 95% CI = [−0.19, −0.03]), suggesting that moral identity negatively moderated both the direct effect of future self-continuity on psychological well-being and its impact on meaning in life. Hypotheses 3 and 4 are not supported. The moderating effect of moral identity is in the opposite direction to the expected positive one.

To assess the practical magnitude of these moderating effects, we examined the change in *R*^2^ (Δ*R*^2^) and computed Cohen’s *f*^2^ when adding the interaction term. For meaning in life, adding the interaction term explained an additional 0.27% of the variance (Δ*R*^2^ = 0.0027, *f*^2^ = 0.005). For psychological well-being, it explained an additional 0.71% of the variance (Δ*R*^2^ = 0.0071, *f*^2^ = 0.009). These results suggest that although the moderating role of moral identity is statistically significant, its practical magnitude is minimal.

Furthermore, following Hayes’ (2018) procedure for testing moderated mediation, evidence for moderated mediation is obtained when the confidence interval for the conditional indirect effect excludes zero. The analysis of conditional indirect effects (refer to Table 4) revealed that the indirect effect of future self-continuity on psychological well-being via meaning in life was 0.11 (95% CI = [0.07, 0.16]) at low moral identity levels, 0.10 (95% CI = [0.06, 0.15]) at moderate levels, and 0.09 (95% CI = [0.05, 0.14]) at high levels. The index of moderated mediation was −0.02 (95% CI = [−0.04, −0.01]), which does not contain zero, indicating that the indirect effect significantly differs across levels of moral identity. To further quantify the conditional mediating effect across different levels of moral identity, we calculated the proportion of the indirect effect relative to the total effect. At low moral identity, the indirect effect accounted for 43.6% of the total effect; at moderate moral identity, 52.9%; and at high moral identity, 70.6%. These results indicate that although the absolute magnitude of the indirect effect decreased slightly as moral identity increased, the proportion of the total effect that was mediated actually increased, suggesting that the mediating role of meaning in life becomes relatively more important for individuals with higher moral identity.

**Figure 2:**
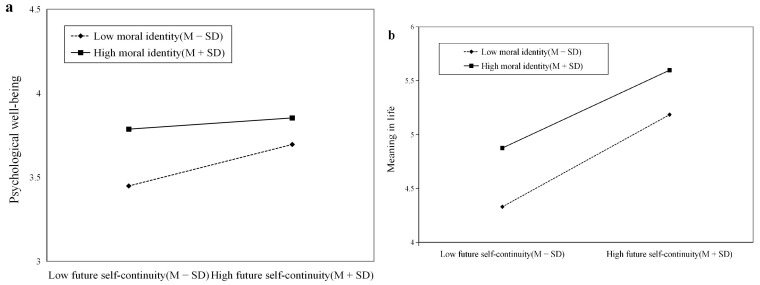
(**a**) Simple slope analysis of the moderating role of moral identity on the relationship between future self-continuity and psychological well-being; (**b**) Simple slope analysis of the moderating role of moral identity on the relationship between future self-continuity and meaning in life.

To further illuminate the specific pattern of this moderating effect, we conducted simple slope analyses. As illustrated in Figure 2a, when college students’ moral identity was at one standard deviation above the mean, future self-continuity was not a significant predictor of psychological well-being (*β* = 0.04, *p* = 0.255, 95% CI = [−0.03, 0.11]). When moral identity was one standard deviation below the mean, future self-continuity positively predicted psychological well-being (*β* = 0.15, *p* < 0.001, 95% CI = [0.07, 0.22]). The findings indicate that moral identity weakens the link between future self-continuity and psychological well-being. Future self-continuity significantly affects psychological well-being in students with low moral identity, but not in those with high moral identity.

Figure 2b demonstrates that moral identity negatively moderated the impact of future self-continuity on meaning in life. Future self-continuity more strongly predicted meaning in life when moral identity was one standard deviation below the mean (*β* = 0.51, *p* < 0.001, 95% CI = [0.44, 0.57]), compared to when moral identity was one standard deviation above the mean, where the effect was notably weaker (*β* = 0.42, *p* < 0.001, 95% CI = [0.36, 0.49]). The findings indicate that future self-continuity significantly influences the meaning in life for college students with low moral identity more than for those with high moral identity.

#### 3.3.1. Dimension-Level Moderated Mediation Analyses

As described in the Procedure and Data Processing section, we conducted separate moderated mediation analyses for each combination of meaning in life sub-dimensions and moral identity sub-dimensions. All analyses used PROCESS Model 8 (Hayes, 2018), controlling for gender and age. Detailed regression results are presented in the Supplementary Materials (Tables S1–S4). The key findings are summarized below.

#### 3.3.2. Presence of Meaning (MILP) as Mediator

When internalization served as the moderator, the interaction between Future self- continuity (FS) and internalization was significantly negative for MILP (*β* = −0.10, *p* = 0.033, 95% CI = [−0.19, −0.01]) and significantly positive for psychological well-being (PWB) (*β* = 0.09, *p* = 0.044, 95% CI = [0.01, 0.17]). The conditional indirect effect of FS on PWB via MILP was 0.143 (95% CI = [0.092, 0.194]) at low internalization and 0.120 (95% CI = [0.076, 0.166]) at high internalization. The index of moderated mediation (IMM) was −0.021 (95% CI = [−0.047, 0.002]), which includes zero. Therefore, internalization does not significantly moderate the indirect effect through MILP. When symbolization served as the moderator, the interaction between FS and symbolization did not significantly predict MILP (*β* = 0.02, *p* = 0.390, 95% CI = [−0.03, 0.08]) but significantly negatively predicted PWB (*β* = −0.15, *p* < 0.001, 95% CI = [−0.20, −0.09]). The conditional indirect effect was 0.083 (95% CI = [0.051, 0.119]) at low symbolization and 0.088 (95% CI = [0.054, 0.129]) at high symbolization. The IMM was 0.004 (95% CI = [−0.006, 0.021]), also including zero. Thus, the indirect effect via MILP was not significantly moderated by symbolization either.

#### 3.3.3. Search for Meaning (MILS) as Mediator

When internalization served as the moderator, the interaction between FS and internalization significantly predicted MILS in the negative direction (*β* = −0.31, *p* < 0.001, 95% CI = [−0.43, −0.19]) and significantly predicted PWB in the positive direction (*β* = 0.12, *p* = 0.007, 95% CI = [0.03, 0.21]). The conditional indirect effect of FS on PWB via MILS was 0.108 (95% CI = [0.067, 0.153]) at low internalization and 0.052 (95% CI = [0.021, 0.092]) at high internalization. The index of moderated mediation (IMM) was −0.052 (95% CI = [−0.087, −0.022]), which does not contain zero, demonstrating a significant moderated mediation. When symbolization served as the moderator, the interaction between FS and symbolization significantly predicted MILS in the negative direction (*β* = −0.11, *p* = 0.003, 95% CI = [−0.18, −0.04]) and also significantly negatively predicted PWB (*β* = −0.13, *p* < 0.001, 95% CI = [−0.18, −0.08]). The conditional indirect effect was 0.086 (95% CI = [0.051, 0.124]) at low symbolization and 0.062 (95% CI = [0.034, 0.095]) at high symbolization. The IMM was −0.020 (95% CI = [−0.034, −0.006]), also significant, indicating that symbolization significantly moderates the indirect effect via MILS. 

Taken together, the negative moderating role of moral identity on the benefits of future self-continuity operates specifically through the search for meaning (MILS) pathway, whereas the presence of meaning (MILP) pathway remains stable across levels of moral identity. Both internalization and symbolization significantly reduce the positive effect of future self-continuity on MILS, and symbolization additionally weakens the direct effect on well-being. These results highlight the importance of distinguishing sub-dimensions in understanding how moral identity interacts with future self-continuity.

## 4. Discussion

### 4.1. The Influence of Future Self-Continuity on Psychological Well-Being

The study found a positive relationship between higher future self-continuity and enhanced psychological well-being. This observation is consistent with previous research (Reiff et al., 2020; Dai et al., 2023) and further highlights the significant value of future self-continuity as a positive psychological resource, thereby providing support for Hypothesis 1. The mechanisms by which future envisioning enhances the present can be interpreted from various perspectives. Future self-continuity is rooted in Markus and Nurius’s (1986) possible selves theory, which suggests that individuals hold various cognitive representations of their future selves: the “hoped-for self” (aspired self), the “expected self” (anticipated self), and the “feared self” (dreaded self). High future self-continuity suggests that individuals are capable of mentally simulating their “future self”, particularly the desired and positively anticipated “hoped-for self” in a vivid, concrete, and imagistic manner. Through this process, the remote possibilities of future events are internalized into one’s current self-concept, thereby offering clear life goals and directions for personal development (Oyserman et al., 2006).

From a motivational perspective, hope theory maintains that a clear cognition of a positive future and the perceived ability to generate pathways toward it constitute the core of hope (Snyder, 2002). Future self-continuity clarifies one’s ‘hoped-for self’, enhancing hope, motivation, and the pursuit of life’s meaning, which in turn elevates psychological well-being. Moreover, the future self-continuity theory proposes that the strength of an individual’s perceived psychological connection with their future self is crucial for prospective motivation. This connection integrates future benefits into present decision-making, essentially serving as a cognitive and emotional preparation for investing in one’s future (Hershfield et al., 2011). Previous research has confirmed that future self-continuity enables individuals to integrate their “present self” and “future self” into a coherent life narrative (Chu & Lowery, 2024). Envisioning future activities strengthens adolescents’ connection between their future and present selves, fostering a stable self-identity linked to a meaningful life (Xue et al., 2024). This crucial psychological resource aids in integrating life experiences, sustaining a positive self-concept (Landau et al., 2009; Sokol & Serper, 2019), and boosting interest in challenges and motivation for goal pursuit (Sims et al., 2020; Adelman et al., 2017). It helps individuals preserve self-continuity amid present and future uncertainties (Lampinen et al., 2004), mitigating negative emotions like anxiety, stress, or depression caused by disruptions in self-continuity and easing feelings of disconnection from their future selves (Sedikides et al., 2023).

Therefore, future self-continuity is not merely a cognitive resource conducive to long-term decision-making; it is also a psychological construct capable of reshaping the sources of motivation. It extends individuals’ psychological horizons from the present to the future, transforming behavioral motivation from reliance on external rewards to the pursuit of inner potential and personal development. This is a crucial mechanism through which individuals attain psychological well-being.

### 4.2. The Mediating Effect of Meaning in Life

This study highlights the crucial mediating effect of meaning in life in linking future self-continuity with psychological well-being. Higher future self-continuity was associated with an enhanced sense of meaning in life, leading to improved psychological well-being. Hypothesis 2 was confirmed, as future self-continuity initiates the process by aiding in the construction of meaning in life. According to meaning-making theory, individuals actively construct meaning from life experiences using language, narrative, and cognitive processing, rather than passively discovering it (Baumeister, 1991). Individuals with strong future self-continuity demonstrate an enhanced ability for mental time travel, allowing them to weave life experiences from various time points into a cohesive, goal-oriented self-narrative, which significantly enriches their sense of meaning in life (Yuan et al., 2025). Within this narrative, the “future self” serves a core orienting function, establishing the direction of the story’s development and its ultimate goals. Consequently, all current experiences, choices, and actions of the individual are endowed with a sense of progression toward these envisioned objectives.

Furthermore, individuals with high future self-continuity possess a heightened capacity for vivid mental simulation of their future (Moss & Wilson, 2018). Research by Waytz et al. (2015) has demonstrated that mental simulation of the future constitutes a significant source of meaning in life. Future self-continuity can anchor individuals in a purpose-driven framework focused on future possibilities, enhancing their perceived meaning in life by constructing a coherent self-narrative that integrates past, present, and future experiences. This enriched sense of meaning further supports psychological well-being by fulfilling fundamental psychological needs. Based on self-determination theory, a well-defined future narrative systematically satisfies the three fundamental psychological needs that contribute to meaning in life. A clear sense of meaning in life enhances behavior alignment with intrinsic values, fulfilling the autonomy need (Ryan & Deci, 2000). In the process of pursuing meaningful goals, individuals continuously learn and overcome challenges, experiencing growth in their capabilities and fulfilling the need for competence (Ryan & Sapp, 2007). When goals encompass altruistic or socially contributive elements, the need for relatedness is also satisfied (Steger et al., 2008a). Extensive research indicates that fulfilling fundamental psychological needs through meaningful activities is crucial for improving long-term well-being (Martela & Ryan, 2016; Weinstein et al., 2013).

College students are in a critical period for forming their worldviews, life philosophies, and values. According to positive psychology, consciously fostering hope for the future is essential for positive development throughout adolescence (Verdugo & Sánchez-Sandoval, 2018). A strong sense of meaning in life directly predicts enhanced mental health (Kleftaras & Psarra, 2012) and life satisfaction (Wolfram, 2023), while also facilitating deeper well-being oriented toward personal growth (Dezutter et al., 2013; Yalçın & Malkoç, 2015). The mediating pathway revealed in this study essentially reflects a two-stage mechanism involving cognitive-narrative integration and motivational-need fulfillment, whereby future self-continuity first constructs a meaningful life narrative framework, which in turn enhances well-being by satisfying basic psychological needs. This finding offers a mechanistic explanation for the transformation of temporal cognition into the experience of well-being, and illuminates a pathway for future-oriented and meaning-seeking interventions targeting college students.

### 4.3. The Moderating Effect of Moral Identity

Contrary to the predicted direction of Hypotheses 3 and 4, moral identity did not strengthen the positive effects of future self-continuity on meaning in life and psychological well-being. Instead, it exhibited a stable and consistent negative moderating effect on both pathways. This result further highlights the boundary effect of moral identity. As an internal value resource, moral identity does not always work synergistically with future self-continuity to promote individuals’ psychological well-being; rather, it may attenuate the positive influence of future self-continuity under certain conditions. Specifically, the higher the level of moral identity, the weaker the positive predictive effect of future self-continuity on meaning in life. Conditional indirect effect analyses further showed that the indirect effect of future self-continuity on psychological well-being via meaning in life was stronger among individuals with low moral identity and not significant among those with high moral identity. Dimension-level analyses showed that the indirect effect via search for meaning was significantly moderated by both internalization and symbolization, whereas the indirect effect via the presence of meaning was not. Notably, the moderating effect of internalization (IMM = −0.052) was larger than that of symbolization (IMM = −0.020). This difference is theoretically meaningful: internalization reflects the centrality of moral traits to one’s self-concept, which may more strongly influence intrapsychic meaning-seeking processes; symbolization pertains to public behavioral expression, which may play a smaller role in the internal pathway from future self-continuity to search for meaning. These findings illustrate the complex interplay between different internal self-resources, namely, the time-based future self and the value-based moral self in the construction of well-being, thereby providing important theoretical implications.

From a resource substitution perspective, moral identity theory (Aquino & Reed, 2002) and moral schema theory (Lapsley & Narvaez, 2004) suggest that individuals with a strong moral identity integrate moral principles into their self-concept, forming a stable “moral self-schema.” This stable moral self inherently supplies individuals with a continuous and steady sense of meaning in life and value direction. Longitudinal evidence suggests that moral identity contributes to the development of meaning in life through multiple pathways. Participants who completed questionnaires measuring presence of meaning and moral identity in their final high school year and two years later showed that moral identity levels significantly predicted perceived meaning growth over the two-year period (Han et al., 2019). Moreover, moral identity is linked to a stronger sense of responsibility and calling (Hardy et al., 2014). This implies that moral identity, as a stable internal value system, may have a developmental function in facilitating the construction of meaning in life. For those with a strong moral identity, a consistent moral self offers an alternative source of meaning and happiness, reducing dependence on external future narratives. Conversely, for individuals whose moral identity is still developing or relatively low, their internal anchors for meaning in life might be less firmly established. Future self-continuity, as a powerful cognitive capacity for temporal reasoning and mental simulation (Waytz et al., 2015), thus emerges as an important psychological resource for exploring meaning in life and constructing life narratives.

Additionally, moral identity may also function as a “double-edged sword.” Meta-analytic evidence indicates that while moral identity is positively associated with moral emotion, like pride and elevation, it also correlates with self-evaluative negative emotions including guilt and shame (Lefebvre & Krettenauer, 2019). Moral identity not only predicts moral behavior (Hertz & Krettenauer, 2016) but also leads to negative emotions like frustration, disappointment, and anxiety due to increased self-monitoring and self-censure (Bandura et al., 1996; Rothmund & Baumert, 2014). Research suggests that the centrality of morality to self-concept amplifies guilt and shame (Kingsford, 2018), with high moral identity individuals reporting stronger experiences of both guilt (Ding et al., 2016) and shame (Abraham & Berline, 2015). When such individuals envision a future self aligned with moral ideals, this process may simultaneously inspire hope and trigger anticipatory anxiety about potential failures to meet those standards, creating an emotional burden that partially offsets the positive effects of future self-continuity.

Furthermore, within the Chinese collectivist cultural context, which stresses self-reflection and sensitivity to others’ evaluations, the moral self is closely bound up with social expectations. This cultural background often heightens moral sensitivity to emotions such as shame and the accompanying pressure for self-monitoring (Laham et al., 2010; Maitner et al., 2017), which may consequently reduce individuals’ psychological well-being. Consequently, individuals with a strong moral identity may exhibit a more intricate well-being profile. While they gain a deep sense of meaning and integration from moral fulfillment, they are also more prone to emotional distress related to moral standards. These future-oriented negative moral emotions might, to some extent, impose a cognitive and emotional burden, partially counterbalancing the positive effects brought by future self-continuity through hope and meaning, ultimately diminishing its facilitating role in promoting well-being.

## 5. Limitations and Prospects

Although the current study advances theoretical integration and mechanism elucidation, several limitations regarding sample representativeness, research design, measurement methods and cultural applicability warrant further examination and extension in future research. First, this study focused exclusively on Chinese college students, and the sample had a disproportionately high proportion of female participants. Prior studies have found that future self-continuity varies with age, with older adults generally showing greater levels (Löckenhoff & Rutt, 2017). Women generally exhibit stronger moral identity than men (Kennedy et al., 2017). Future research should broaden to encompass various age groups, occupational categories, and a more balanced gender distribution to evaluate the generalizability of the current findings.

Second, the cross-sectional design employed precludes strict causal inferences regarding the relationships among variables. Future studies might utilize longitudinal designs, such as collecting data in three waves at six-month or annual intervals, to explore long-term causal links (Zhang et al., 2025). Alternatively, experimental approaches, like manipulating future self-continuity or priming varying levels of moral identity, could be employed to further confirm the causal relationships identified in this study. Additionally, while statistical tests did not indicate significant common method bias, potential biases like social desirability effects cannot be completely dismissed. Future research should integrate diverse assessment methods, such as behavioral experiments and physiological measures, to improve the objectivity and validity of the findings.

Furthermore, the theoretical model was developed within the Chinese sociocultural context. Given the profound cultural differences in self-construal, for instance, North Americans typically exhibit stronger independent selves, whereas East Asians tend toward more interdependent selves (Ji et al., 2022), future research should explore how cultural values (individualism/collectivism) shape the interactive patterns between moral identity and future self-continuity. Additionally, future studies could investigate whether other personality traits or environmental factors (e.g., perfectionism, social support) buffer the potential emotional burden associated with high moral identity, thereby constructing a more comprehensive model of the mechanisms underlying well-being.

## 6. Conclusions

The current study examined a moderated mediation model to explain how future self-continuity affects psychological well-being. These results showed that future self- continuity significantly enhanced psychological well-being, with meaning in life partially mediating this relationship. Moral identity negatively moderated the effect of future self- continuity on both meaning in life and psychological well-being. Specifically, the beneficial effects were stronger among individuals with low moral identity. Thus, interventions aimed at enhancing well-being should not only strengthen future self- continuity but also attend to the potential emotional burden associated with high moral identity.

## Figures and Tables

**Table 1:** Descriptive statistics and correlations for study variables (N = 890).

	*M*	*SD*	1	2	3	4	5	6
1 Sex	1.64	0.48	1					
2 Age	19.04	1.12	0.01	1				
3 Future self-continuity	4.78	0.85	0.04	0.02	1			
4 Meaning in life	4.84	0.81	−0.03	0.02	0.64 ***	1		
5 Moral Identity	3.48	0.47	0.07 *	0.03	0.54 ***	0.55 ***	1	
6 Psychological Well-Being	4.22	0.67	0.04	−0.04	0.38 ***	0.44 ***	0.38 ***	1

Note: * *p* < 0.05; *** *p* < 0.001.

**Table 2:** Analysis of the mediation model test of meaning in life.

	Dependent Variable: Meaning in Life				Dependent Variable: Psychological Well-Being			
	*β*	*SE*	*t*	95% CI	*β*	*SE*	*t*	95% CI
Sex	−0.10	0.04	−2.41 *	[−0.19, −0.02]	0.07	0.04	1.59	[−0.02, 0.15]
Age	0.01	0.02	0.42	[−0.03, 0.04]	−0.03	0.02	−1.69	[−0.07, 0.01]
Future self-continuity	0.61	0.02	24.73 ***	[0.56, 0.66]	0.13	0.03	4.08 ***	[0.07, 0.19]
Meaning in life					0.28	0.03	8.71 ***	[0.22, 0.35]
R^2^	0.41				0.21			
F	204.75 ***				59.22 ***			

Note: *β* = unstandardized regression coefficient; 95% CI = confidence interval for *β*. * *p* < 0.05; *** *p* < 0.001.

**Table 3:** Analysis of the moderated mediating model of future self-continuity on psychological well-being.

	Dependent Variable: Meaning in Life				Dependent Variable: Psychological Well-Being			
	*β*	*SE*	*t*	95% CI	*β*	*SE*	*t*	95% CI
Sex	−0.14	0.04	−3.35 **	[−0.22, −0.06]	0.03	0.04	0.83	[−0.05, 0.12]
Age	0.01	0.02	0.27	[−0.03, 0.04]	−0.03	0.02	−1.76	[−0.07, 0.01]
Future self-continuity	0.75	0.14	5.32	[0.48, 1.03]	0.48	0.14	3.37	[0.20, 0.77]
Moral Identity	0.91	0.20	4.60	[0.52, 1.30]	0.80	0.20	4.01	[0.41, 1.20]
Future self-continuity × Moral Identity	−0.08	0.04	−2.12 *	[−0.16, −0.01]	−0.11	0.04	−2.86 **	[−0.19, −0.03]
Meaning in life					0.22	0.03	6.65 ***	[0.16, 0.29]
R^2^	0.47				0.24			
F	158.42 ***				45.87 ***			

Note: *β* = unstandardized regression coefficient; 95% CI = confidence interval for *β*. * *p* < 0.05; ** *p* < 0.01; *** *p* < 0.001.

**Table 4:** Analysis of Indirect Effects of Conditions.

Moral Identity	Mediating Effect	Boot SE	Boot LLCI	Boot ULCI
Low	0.11	0.02	0.07	0.16
Middle	0.10	0.02	0.06	0.15
High	0.09	0.02	0.05	0.14

LL = low limit, CI = confidence interval, UL = upper limit.

## Data Availability

The datasets generated during and/or analyzed during the current study are available from the corresponding author on reasonable request.

## Supplementary Materials

The following supporting information can be downloaded at: https://www.mdpi.com/article/10.3390/bs16050647/s1, Table S1: Dimension-level moderated mediation analyses with presence of meaning as mediator and internalization as moderator; Table S2: Dimension-level moderated mediation analyses with presence of meaning as mediator and symbolization as moderator; Table S3: Dimension-level moderated mediation analyses with search for meaning as mediator and internalization as moderator; Table S4: Dimension-level moderated mediation analyses with search for meaning as mediator and symbolization as moderator.
