# Supplementary material for: Future Self-Continuity and Psychological Well-Being in Chinese College Students: The Mediating Role of Meaning in Life and the Moderating Role of Moral Identity
Source: Behav Sci (Basel). 2026 Apr 26;16(5):647. doi: 10.3390/bs16050647 (PMC13203122; doi:10.3390/bs16050647)
Supplement: Supplementary file 1 [file behavsci-16-00647-s001.zip › behavsci-4226972-supplementary.pdf]

Future Self-Continuity and Psychological Well-Being in Chinese College Students: The Mediating Role of Meaning in Life and the Moderating Role of Moral Identity

Supplementary Materials

Table S1. Dimension-level moderated mediation analyses with presence of meaning as mediator and internalization as moderator

	Dependent variable: Presence of meaning				Dependent variable: Psychological well-being			
	β	*SE*	*t*	*95% CI*	*β*	*SE*	*t*	*95% CI*
Sex	-0.15	0.05	-3.21**	[-0.24, -0.06]	0.01	0.04	0.35	[-0.07, 0.09]
Age	0.01	0.02	0.17	[-0.03, 0.04]	-0.03	0.02	-1.85	[-0.07, 0.01]
Future self-continuity	0.99	0.17	5.92***	[0.67, 1.33]	-0.21	0.15	-1.33	[-0.51, 0.09]
Internalization	0.46	0.24	1.92	[-0.01, 0.92]	-0.09	0.21	-0.41	[-0.51, 0.33]
Future self-continuity ×Internalization	-0.10	0.05	-2.14*	[-0.19, -0.01]	0.09	0.04	2.02*	[0.01, 0.17]
Presence of meaning					0.21	0.03	6.83***	[0.15, 0.27]
R^2	0.41				0.25			
F	123.41***				48.44***			

Note： *β* = unstandardized regression coefficient; 95% CI = confidence interval for *β*.
**p* < 0.05; ***p* < 0.01; ****p* < 0.001

Table S2. Dimension-level moderated mediation analyses with presence of meaning as mediator and symbolization as moderator

	Dependent variable: Presence of meaning				Dependent variable: Psychological well-being			
	β	*SE*	*t*	*95% CI*	*β*	*SE*	*t*	*95% CI*
Sex	-0.12	0.04	-2.69**	[-0.20, -0.03]	0.03	0.04	0.80	[-0.05, 0.12]
Age	0.01	0.02	0.14	[-0.03, 0.04]	-0.03	0.02	-1.46	[-0.06, 0.01]
Future self-continuity	0.37	0.10	3.65***	[0.17, 0.57]	0.71	0.10	7.06***	[0.51, 0.91]
symbolization	0.35	0.14	2.46*	[0.07, 0.63]	0.79	0.14	5.59***	[0.51, 1.06]
Future self-continuity ×symbolization	0.02	0.03	0.86	[-0.03, 0.08]	-0.15	0.03	-5.68***	[-0.20, -0.09]
Presence of meaning					0.19	0.03	5.76***	[0.13, 0.26]
R^2	0.49				0.21			
F	167.05***				38.58***			

Note： *β* = unstandardized regression coefficient; 95% CI = confidence interval for *β*.
**p* < 0.05; ***p* < 0.01; ****p* < 0.001

Table S3. Dimension-level moderated mediation analyses with search for meaning as mediator and internalization as moderator

	Dependent variable: search for meaning				Dependent variable: Psychological well-being			
	β	*SE*	*t*	*95% CI*	*β*	*SE*	*t*	*95% CI*
Sex	-0.11	0.06	-1.93	[-0.23, 0.01]	0.01	0.04	0.07	[-0.08, 0.08]
Age	0.01	0.03	0.24	[-0.04, 0.05]	-0.03	0.02	-1.88	[-0.07, 0.01]
Future self-continuity	1.56	0.21	7.33***	[1.14, 1.98]	-0.26	0.16	-1.68	[-0.57, 0.04]
Internalization	1.89	0.30	6.27***	[1.29, 2.48]	-0.31	0.22	-1.42	[-0.74, 0.12]
Future self-continuity ×Internalization	-0.31	0.06	-5.06***	[-0.43, -0.19]	0.12	0.04	2.69**	[0.03, 0.21]
search for meaning					0.17	0.02	7.02***	[0.12, 0.21]
R^2	0.31				0.25			
F	78.39***				49.01***			

Note: *β* = unstandardized regression coefficient; 95% CI = confidence interval for *β*.
$^*p < 0.05$; $^{**}p < 0.01$; $^{***}p < 0.001$

Table S4. Dimension-level moderated mediation analyses with search for meaning as mediator and symbolization as moderator

	Dependent variable: search for meaning				Dependent variable: Psychological well-being			
	β	*SE*	*t*	*95% CI*	*β*	*SE*	*t*	*95% CI*
Sex	-0.04	0.06	-0.65	[-0.15, 0.08]	0.02	0.04	0.45	[-0.06, 0.10]
Age	0.01	0.02	0.60	[-0.03, 0.06]	-0.03	0.02	-1.59	[-0.06, 0.01]
Future self-continuity	0.80	0.14	5.83***	[0.53, 1.07]	0.64	0.10	6.35***	[0.44, 0.83]
symbolization	1.01	0.19	5.27***	[0.64, 1.39]	0.67	0.14	4.78***	[0.40, 0.95]
Future self-continuity ×symbolization	-0.11	0.04	-3.03**	[-0.18, -0.04]	-0.13	0.03	-4.80***	[-0.18, -0.08]
search for meaning					0.18	0.02	7.32***	[0.13, 0.23]
R^2	0.31				0.23			
F	78.93***				42.734***			

Note: *β* = unstandardized regression coefficient; 95% CI = confidence interval for *β*.
$^*p < 0.05$; $^{**}p < 0.01$; $^{***}p < 0.001$
